# Association between sarcopenia and cognitive function in older Chinese adults: Evidence from the China health and retirement longitudinal study

**DOI:** 10.3389/fpubh.2022.1078304

**Published:** 2023-01-10

**Authors:** Hongzhen Du, Miao Yu, Hongmei Xue, Xuning Lu, Yaping Chang, Zengning Li

**Affiliations:** ^1^Department of Nutrition, The First Hospital of Hebei Medical University, Shijiazhuang, China; ^2^Hebei Key Laboratory of Nutrition and Health, Shijiazhuang, Hebei, China

**Keywords:** sarcopenia, possible sarcopenia, cognitive function, muscle mass, older adults

## Abstract

**Background:**

Sarcopenia and cognitive impairment are the most common causes of disability in the aging population. The potential role of sarcopenia in the development of cognitive impairment remains poorly understood. A cross-sectional analysis was performed using nationally representative data to evaluate associations between sarcopenia and cognition in China.

**Methods:**

We included 2,391 participants (35.63% female) who were at least 60 years of age in 2015 from the China Health and Retirement Longitudinal Study (CHARLS). Muscle strength, appendicular skeletal mass (ASM), and physical performance measurements, were measured to diagnose sarcopenia according to the Asian Working Group for Sarcopenia 2019 (AWGS2019). Cognitive function was assessed by 10 items in the Telephone Interview for Cognitive Status (TICS-10), delayed word recall, and graph drawing. Based on cognitive score tertiles, data were divided into three groups. Multiple linear and logistic regression models were used to assess the relationship between sarcopenia and cognition.

**Results:**

The prevalence of possible sarcopenia was 27.16% for men and 27.46% for women. Cognitive decline was significantly associated with sarcopenia status (β = −0.88, *p* < 0.001) and negatively associated with components of sarcopenia in male group. The results remained consistent in male after further adjusting for creatinine, uric acid, blood sugar, etc. Low cognitive function in female was only associated with low muscle strength (β = −0.85, *p* = 0.02). In addition, participants with possible sarcopenia had greater risk of cognitive decline than those without sarcopenia (OR = 1.41; 95% CI: 1.06–1.87). However, the same association was not significant in female group.

**Conclusion:**

We suggest that sarcopenia might be associated with cognition function, with possible sarcopenia being significantly associated with higher cognition risk in China population, which providing a further rationale for timely recognition and management of sarcopenia.

## Background

Sarcopenia is a skeletal muscle disease rooted in the lifelong accumulation of adverse muscle changes due to increased protein catabolism or increased anabolic resistance, both common conditions in older adults, and it can increase the incidence of poor clinical outcomes, such as falls, fractures, physical disability, and even mortality (1). Studies have shown that 7–10% of people aged 60–70 years and 30% of people over 80 years have sarcopenia (2). Low cognitive function, a neurodegenerative process due to increasing age, is an impairment of functions in multiple domains, including attention, memory, execution, language, literacy, numeracy, reasoning, planning, and orientation (3). It has been suggested that the prevalence of mild cognitive impairment in people aged 60 and over is 15–20% (4). Aging plays an important role in both skeletal muscle degeneration and low cognitive function. Thus, sarcopenia and cognitive decline share a common pathophysiological pathway. The pathophysiological mechanisms of sarcopenia include aging, reduced activity, neuromuscular damage, insulin resistance, hormonal dysregulation, oxidative stress and chronic inflammation (5). Additionally, these predisposing conditions are also linked to cognitive dysfunction (6). It is unclear how skeletal sarcopenia affects cognitive function, but several studies indicate that several myokines are produced by skeletal muscle and secreted, including those regulating mood, learning, motor activity, and neuronal damage protection, indicating the presence of muscle-brain crosstalk (7). In addition, lifestyle factors, such as physical inactivity, poor diet, obesity and smoking, are common risk factors for both diseases. Moreover, sarcopenia may interact with cognitive function. Advanced sarcopenia and its accompanying frailty and loss of independence are clear causes of depression and low cognitive function. Conversely, low cognitive function leads to reduced physical activity and dietary intake, which in turn accelerates sarcopenia.

At present, accumulating evidence suggests that sarcopenia may be associated with an increased risk of cognitive impairment in older adults, although the findings are inconsistent (8, 9). A meta-analysis confirmed that sarcopenia and cognitive dysfunction were positively associated (10), but these results remained inconsistent in subgroup analyses by study population, study region. China has the largest older population in the world. The total population in China was 1.38 billion, of which adults aged 60 years old or over represented 16.7% as at the end of 2016 (11). A systematic review has pooled the estimate of sarcopenia prevalence in community-dwelling Chinese older adults (male: 12.9%; female: 11.2%) (12), and around 10% of older adults have cognitive impairment (13). However, to our knowledge, relatively few studies based on a large Chinese population have explored the relationship between sarcopenia and its components with cognitive dysfunction in the older population (14, 15). Nationally representative data from the China Health and Retirement Longitudinal Study (CHARLS) was used to conduct a cross-sectional analysis to explore the relationship between sarcopenia status and cognitive function in Chinese communities.

## Materials and methods

### Study population

The CHARLS is a longitudinal study of people over the age of 45 in China. This study was first established in 2011 and is an ongoing nationally representative longitudinal survey that aims to explore the socioeconomic determinants and consequences of aging (16). Briefly, high quality data from CHARLS was collected through one-on-one interviews using a structured questionnaire. Multilevel stratified probability-proportional-to-size sampling strategy was employed in recruiting the study participants. Sociodemographic, lifestyle, and health data were collected using standardized questionnaires. In 2011, 17,708 participants within 10,257 families were interviewed in 150 counties (districts) and 450 villages in 28 provinces in China. In order to ensure representativeness of the data, these data include weighting variables. Follow-up surveys were conducted every 2 years after the baseline survey (16).

**Figure 1:**
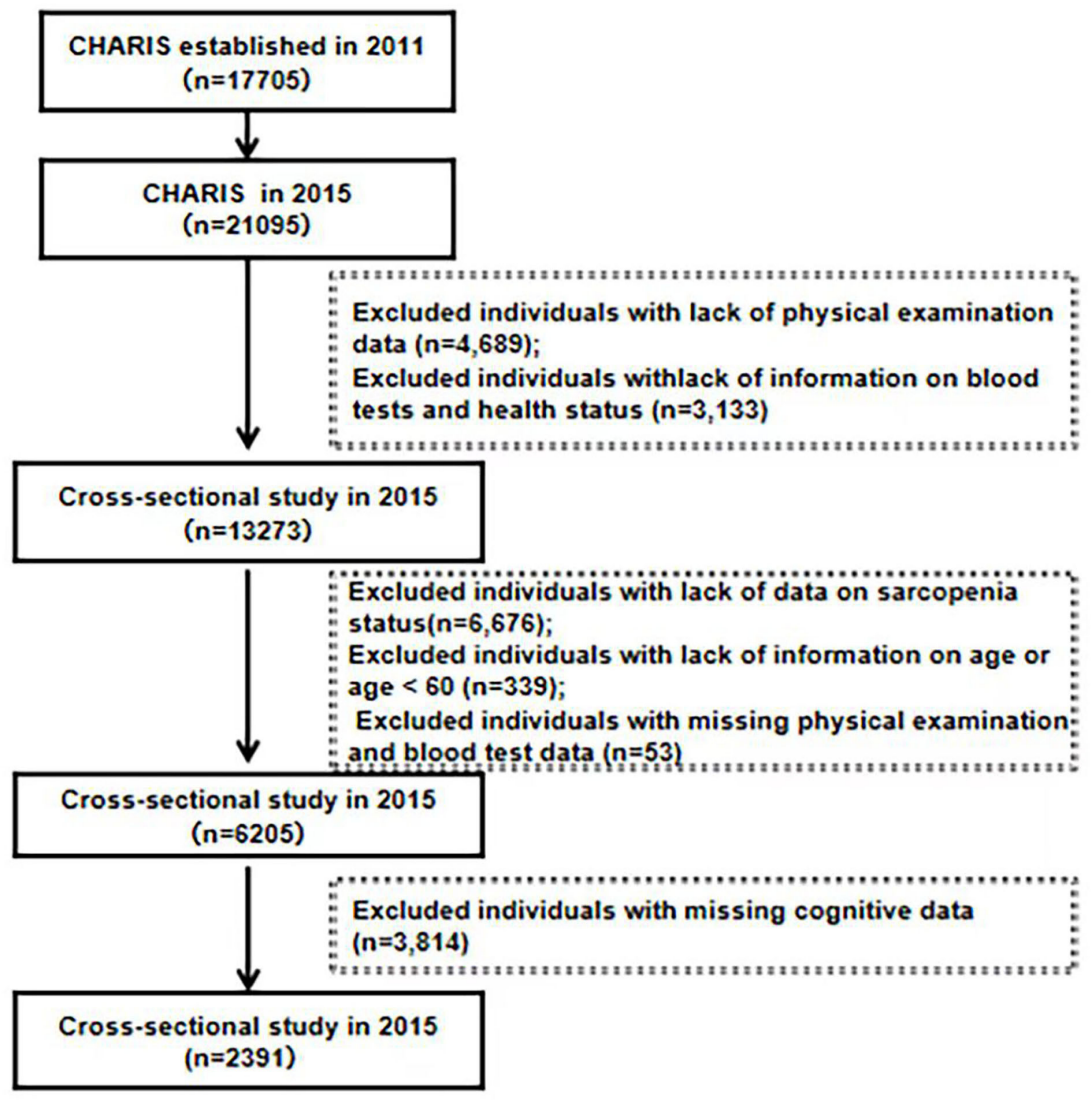
Flowchart for the study sample. HS, handgrip strength; GS, gait speed; ASM, appendicular skeletal muscle mass, CST, chair stand text.

In this study, CHARLS data from 2015 were used. The inclusion criteria for this study were as follows: (a) individuals who were at least 60 years of age at the time of the 2015 CHARLS study and (b) individuals with available data on their sarcopenia status. The exclusion criteria were as follows: individuals with (a) missing sarcopenia status data and cognitive data; (b) missing age data; (c) age <60 years; (d) no physical examination data or blood data. In total, 21,095 participants were interviewed by the CHARLS in 2015. From the CHARLS data booklet we can also derive a blood response rate of only 64%, which may be due to the fact that the collection of blood samples was invasive. In addition, the purpose of the CHARLS data study was to provide high quality data on households and individuals aged 45 years and older in China, so we found 7,222 individuals younger than 60 years of age, and we had to exclude data from these groups as well. During our screening stages, some of the participants were excluded due to a lack of physical examination data (*n* = 4,689), lack of information on blood tests and health status (*n* = 3,133), lack of data on sarcopenia status (*n* = 6,676), lack of information on age or age <60 (*n* = 339), missing physical examination and blood test data (*n* = 53), and missing cognitive data (*n* = 3,814). Thus, a total of 2,391 participants were included the cross-sectional analysis. The specific screening process is shown in Figure 1. The data were obtained through application from the National School of Development of Peking University (China Economic Research Center). Since this study was a secondary analysis of CHARLS data, we did not require a separate ethical approval.

### Assessment of sarcopenia status

The recommended diagnostic methods of AWGS 2019 were used in the present study (17). The AWGS 2019 guidelines define “possible sarcopenia” as the presence of either low muscle strength or low physical performance. Definition of Sarcopenia is diagnosed when low muscle mass plus low muscle strength, or low physical performance. Severe sarcopenia is considered when low levels of muscle strength, muscle mass and physical performance are detected. Participants without any low muscle strength, low muscle mass, or low physical performance were defined as no sarcopenia.

### Muscle strength

Measurements of grip strength were used to determine the overall the strength of muscle. The grip strength of both the dominant and non-dominant hands was measured three times, with the participant was instructed to squeezing a dynamometer (YuejianTM WL-1000, Nantong Yuejian Physical Measurement Instrument Co., Ltd., Nantong, China) as hard as they could (18). A cut-off point with insufficient grip strength was <18 kg for female and <28 kg for male (17).

### Appendicular skeletal mass (ASM)

In our article, we used an anthropometric equation to estimate the muscle mass, which has previously been validated in Chinese individuals (19, 20). The ASM equation model showed a high level of agreement with DXA. (19, 20):


(1) $$\begin{array}{l} \text{ASM}=0.193{\;}^{*}\text{ body weight}+0.107{\;}^{*}\text{ height}-4.157{\;}^{*}\text{ sex}\\ -0.037{\;}^{*}\text{ age}-2.631 \end{array}$$

where ASM is in kg, height is in cm, weight is in kg, age is in years, and sex is represented by 1 (male) or 2 (female).

Height-adjusted muscle mass was calculated as ASM/Ht^2^ = ASM/height (m)^2^. The cut-off point for low muscle mass was based on the lowest 20% percentile of ASM/Ht^2^ in the study population. Since our data are derived from the 2015 CHARLS data, we refer to the criteria of Wu et al. (21). Therefore, the ASM/Ht2 cut-off for female was <5.08 kg/m^2^, and the ASM/Ht^2^ cut-off for male was <6.88 kg/m^2^.

### Physical performance measurements (physical fitness)

Chair stand tests and gait speed tests were used to measure physical performance. Gait speed (GS) was used to measure the participant's usual walking speed (m/s) over a 2.5-m distance. Participants walked the 2.5-m distance at normal speed, once back and forth (i.e., twice), timed by a stopwatch; the average of the two recorded values was used (22). Repeated chair stands were used to measure the body strength and endurance (23). Test participants sit on a chair with no armrests to begin the test. In the fifth stand up-sit down cycle, timing came to an end when the patients' buttocks reached the chair. It takes a participant to stand up from a chair five times, keeping their arms folded over their chests to measure the chair stand tests. The criteria for low physical performance were gait speed tests that were calculated to be <1 m/s or 5 chair stands that exceeded 12 s in total (17).

### Cognitive function assessment

This study measured four dimensions of cognitive function, including orientation, attention, episodic memory, and visuospatial abilities. Orientation and attention were evaluated by the TICS-10, on a scale of 0 to 10 (9). Attention was assessed using the test in which participants were asked to subtract 7 from 100 five times consecutively. Orientation was assessed by asking the participant the date (month, day, year), day of the week, and season of the year. Episodic memory was measured by immediate and delayed word recall (24). Immediate recall was assessed to asking participants to recall as many words as possible immediately after the interviewer read 10 Chinese nouns. Delayed recall was measured by asking subjects to recall as many original words as possible after 4–10 min. The episodic memory score was calculated by the average number of immediate and delayed word recalls on a scale of 0–10 (25). Visuospatial abilities are assessed through graphic rendering. Respondents were shown a painting and asked to draw a similar figure. Respondents who successfully drew the painting received 1 point, while respondents who failed to draw the painting received 0 points (25). Interviews were conducted face to face to assess the dimensions of cognitive function. The cognitive score including the total score of TICS-10, word recall and graph drawing, ranged from 0–21 (26), with a higher score indicating better cognitive function. Then, the individuals were classified according to tertiles of the cognitive score (Lowest tertile: <11.5; Middle tertile: 11.5–14; Highest tertile: > 14).

### Covariates

Sociodemographic and health-related factors were included as covariates. Sociodemographic variables included age, sex and educational attainment (below elementary school, or primary school and above). Health-related factors consist of body mass index (BMI), smoking (self-reported; yes/no), and blood measurements include blood glucose, total cholesterol (TC), low-density lipoprotein (LDL), creatinine, uric acid, C-reactive protein (CRP) and glycated hemoglobin (GHB).

### Statistical analysis

Categorical data are presented as proportions, and continuous data are reported as means ± standard deviations or medians and tertile range for continuous variables and as percentages for categorical variables. First, based on cognitive score tertiles, data were divided into three groups. The baseline characteristics of the cross-sectional samples were summarized and compared between these groups using chi-square tests, Student's *t*-tests, and analyses of variance (ANOVAs). Second, the subjects were divided into two groups according to the diagnostic criteria: those without sarcopenia and those with possible sarcopenia. Additionally, the subjects were divided according to sex and analyzed separately to determine their baseline characteristics and differences in cognitive scores between the two sarcopenia groups. Multiple linear regression models were performed to analyze the relationship between sarcopenia (and its defining components) with the cognitive scores. And logistic regression analysis was used to calculate odds ratios (ORs) with 95% confidence intervals (CIs). The covariables models adjusted were as follows: Model 1: adjusted for smoking, education and age; Model 2: as model 1 and additionally adjusted for BMI; Model 3: as Model 2 and additionally adjusted for creatinine, uric acid, blood sugar, LDL, TC, CRP, GHB.

## Results

The baseline characteristics of the study subjects according to sex and likelihood of sarcopenia are shown in Table 1. In our study, 652 subjects were included in the possible sarcopenia, 295 in the sarcopenia group, 94 in the severe sarcopenia group. The possible sarcopenia group includes part of the population in the sarcopenia group and the sarcopenia includes part of the population in the severe sarcopenia group. As the three groups are not independent, the subjects only were divided into two groups according to the diagnostic criteria: those without sarcopenia and those with possible sarcopenia. Among the male subjects (*n* = 1,539), 418 (27.16%) had possible sarcopenia, and among the female subjects (*n* = 852), 234 (27.46%) had possible sarcopenia. Regardless of sex, there were significant differences between those with and without possible sarcopenia in terms of age, height, weight, muscle mass, muscle strength, and physical activity (*p* < 0.05). Furthermore, man in the no possible sarcopenia group had higher level of education (*p* < 0.001). The cognitive scores of males with possible sarcopenia (12.00) were significantly lower (*p* < 0.001) than those of males without possible sarcopenia (13.00) in males; the same pattern was observed in females.

**Table 1:** Participant characteristics according to sarcopenia status (*n* = 2,391).

**Variables**	**Overall** **(*n* = 2,391)**	**Male (*****n*** = **1,539)**			**Female (*****n*** = **852)**		
		**No possible sarcopenia** **(*****n*** = **1,121)**	**Possible sarcopenia** **(*****n*** = **418)**	***P*** **-value**	**No possible sarcopenia** **(*****n*** = **618)**	**Possible sarcopenia** **(*****n*** = **234)**	***P-*** **value**
Age	66.00 (7.00)	66.00 (8.00)	69.00 (9.00)	<0.001	65.00 (6.00)	67.00 (9.00)	<0.001
Height (cm)	159.90 (12.00)	164.40 (9.00)	162.50 (9.00)	<0.001	153.00 (8.00)	152.00 (8.00)	0.01
Weight (kg)	60.20 (15.00)	63.00 (15.00)	59.55 (15.00)	<0.001	58.00 (12.00)	55.00 (14.00)	0.01
BMI (kg/m^2^)	23.59 (5.00)	23.35 (5.00)	22.41 (5.00)	<0.001	25.00 (4.25)	24.00 (5.25)	0.08
HS (kg)	30.75 (13.00)	37.00 (9.00)	26.95 (10.00)	<0.001	26.00 (7.00)	19.00 (8.00)	<0.001
GS (m/s)	3.03 (1.00)	2.87 (1.00)	3.33 (1.00)	<0.001	3.00 (0.25)	4.00 (1.00)	<0.001
CST (s)	8.81 (4.00)	7.97 (3.00)	12.13 (5.00)	<0.001	9.00 (3.00)	13.00 (4.00)	<0.001
Waist circumference (cm)	87.00 (15.00)	86.20 (14.00)	86.00 (16.00)	0.3	88.00 (14.00)	88.00 (13.25)	0.6
ASM (kg)	18.40 (6.00)	20.52 (3.00)	19.62 (4.00)	<0.001	14.00 (3.00)	13.00 (3.00)	0.001
Cognitive score	13.00 (4.50)	13.00 (4.00)	12.00 (4.00)	<0.001	13.50 (4.00)	12.50 (4.50)	<0.001
**Smoking**							
Smoking (%)	774 (32.2%)	549 (74.5%)	188 (25.5%)	0.2	21 (56.8%)	16 (43.2%)	0.03
Quit smoking (%)	464 (19.4%)	304 (69.7%)	132 (30.3%)		17 (60.7%)	11 (39.3%)	
Never smoke (%)	1,153 (48.2%)	268 (73.2%)	98 (26.8%)		580 (73.7%)	207 (26.3%)	
**Education**							
Below elementary school (%)	497 (100%)	207 (66.8%)	103 (33.2%)	0.007	128 (67.4%)	59 (31.6%)	0.2
Primary school and above (%)	1,894 (100%)	914 (74.4%)	315 (25.6%)		490 (73.7%)	175 (26.3%)	

BMI, body mass index; HS, hand strength; GS, gait speed; CST, 5 chair stand tests; ASM, appendicular skeletal mass.

Table 2 shows the data separated into three categories based on tertiles of cognitive scores. The group with the highest cognitive scores also performed faster on the 5 chair stands and had a stronger grip and faster gait. Regardless of sex, the group with the highest cognitive scores had a higher ASM (*p* < 0.01). Among male subjects, the percentage of those with the lowest cognitive scores with possible sarcopenia (41.9%) was higher than that of those without possible sarcopenia (32.6%). Comparable results were also observed in females (*p* < 0.05).

**Table 2:** Characteristics of study participants classified by Cognitive score (*n* = 2,391).

**Variables**	**Overall** **(*n* = 2,391)**	**Male (*****n*** = **1,539)**				**Female (*****n*** = **852)**			
		**Lowest tertile** **(*****n*** = **541)**	**Middle tertile** ***(n*** = **511)**	**Highest tertile** **(*****n*** = **487)**	***P*** **-value**	**Lowest tertile** **(*****n*** = **306)**	**Middle tertile** **(*****n*** = **248)**	**Highest tertile** **(*****n*** = **298)**	***P-*** **value**
Age (years)	66.00 (7.00)	67.00 (9.00)	66.00 (8.00)	65.00 (7.00)	<0.001	66.00 (9.00)	65.00 (7.00)	65.00 (7.00)	0.007
Height (cm)	159.90(12.00)	162.30 (9.00)	163.50 (9.00)	165.26 (6.16)	<0.001	152.00 (8.00)	153.00 (7.00)	153.00 (7.00)	<0.001
Weight (kg)	60.20 (15.00)	59.60 (15.00)	62.30 (14.00)	64.90 (15.00)	<0.001	56.64 (9.74)	58.00 (13.00)	58.00 (14.00)	0.06
BMI (kg/m^2^)	23.59 (5.00)	22.63 (5.00)	23.08 (4.00)	23.66 (5.00)	<0.001	24.00 (5.00)	24.00 (4.00)	25.00 (5.00)	1.0
HS (kg)	30.75 (13.00)	33.32 (7.46)	35.05 (10.00)	37.40 (10.00)	<0.001	23.00 (8.00)	24.00 (7.75)	25.00 (7.00)	<0.001
GS (m/s)	3.03 (1.00)	3.18 (1.00)	2.94 (1.00)	2.83 (1.00)	<0.001	3.00 (1.00)	3.00 (1.00)	3.00 (1.00)	<0.001
CST (s)	8.81 (4.00)	8.96 (4.00)	8.62 (4.00)	8.25 (3.00)	0.001	9.00 (3.00)	9.00 (4.00)	9.00 (4.00)	0.004
Waist circumference (cm)	87.00 (15.00)	84.20 (16.00)	86.20 (14.00)	88.10 (14.00)	<0.001	88.00 (13.50)	88.00 (14.75)	88.00 (13.00)	0.5
ASM (kg)	18.40 (6.00)	19.61 (2.61)	20.45 (2.66)	20.98 (3.00)	<0.001	14.00 (3.00)	14.00 (3.00)	14.00 (3.00)	0.002
**Smoking**									
Smoking (%)	774 (32.2%)	272 (36.9%)	237 (32.2%)	228 (30.9%)	0.5	17 (45.9%)	11 (29.7%)	9 (24.3%)	0.02
Quit smoking (%)	464 (19.4%)	149 (34.2%)	154 (35.3%)	133 (30.5%)		11 (39.3%)	14 (50.0%)	3 (10.7%)	
Never smoke (%)	1,153 (48.2%)	120 (32.8%)	120 (32.8%)	126 (34.4%)		278 (35.3%)	223 (28.3%)	286 (36.3%)	
**Education**									
Below elementary school (%)	497 (20.7%)	109 (35.2%)	119 (38.4%)	82 (26.5%)	0.04	69 (36.9%)	56 (29.9%)	62 (33.2%)	0.8
Primary school and above (%)	1,894 (79.3%)	432 (35.2%)	392 (31.9%)	405 (33.0%)		237 (35.6%)	192 (28.9%)	236 (35.5%)	
**Sarcopenia**									
No possible sarcopenia (%)	1,739 (72.7%)	366 (32.6%)	360 (32.1%)	395 (35.2%)	<0.001	208 (33.7%)	177 (28.6%)	233 (37.7%)	0.02
Possible sarcopenia (%)	652 (27.3%)	175 (41.9%)	151 (36.1%)	92 (22.0%)		98 (41.9%)	71 (31.3%)	65 (27.8%)	

BMI, body mass index; HS, hand strength; GS, gait speed; CST, 5 chair stand tests; ASM, appendicular skeletal mass.

Table 3 illustrates the relationships between sarcopenia, its defining components, and cognitive function. Sarcopenia was adversely linked with cognitive scores in the unadjusted model, with regression coefficients of −0.88 (95% CI: −1.19, −0.58) in males and −0.79 (95% CI: −1.23, −0.36) in females. Components of sarcopenia, such as low muscle mass [males: −1.06 (−1.43, −0.70); females: −0.79 (−1.42, −0.15)], low muscle strength [males: −1.15 (−1.52, −0.79); females: −1.37 (−1.95, −0.79)], and low gait speed [males: −0.75 (−1.07, −0.43); females: −0.78 (−1.28, −0.28)], were negatively correlated with cognitive scores (*p* < 0.05). After adjusted for age, education, smoking, BMI, significant negative correlations (correlation coefficient > 0.5) were observed between cognitive function and sarcopenia, cognitive function and low muscle mass, cognitive function and low GS (Table 3). This effect was attenuated after further adjustment, but remained significant in male after additional adjustment for creatinine, uric acid, blood sugar, etc., (Model 3). Low cognitive function in female was only associated with low muscle strength (β = −0.85, *p* = 0.02) in the fully adjusted model (Model 3).

**Table 3:** Multivariate linear regression model of sarcopenia and cognitive function.

**Models**	**Sarcopenia** **(*n* = 652)** **β (95% CI)**	***P*-value**	**Low muscle mass** **(*n* = 337)** **β (95% CI)**	***P*-value**	**Low muscle strength** **(*n* = 366)** **β (95% CI)**	***P-*value**	**Low GS** **(*n* = 1,865)** **β (95% CI)**	***P*-value**
**Male (*****n*** = **1,539)**								
Unadjusted	−0.88 (−1.19, −0.58)	<0.001	−1.06 (−1.43, −0.70)	<0.001	−1.15 (−1.52, −0.79)	<0.001	−0.75 (−1.07, −0.43)	<0.001
Model 1	−0.64 (−0.95, −0.33)	<0.001	−0.77 (−1.15, −0.39)	<0.001	−0.87 (−1.24, −0.50)	<0.001	−0.61 (−0.93, −0.30)	<0.001
Model 2	−0.63 (−0.95, −0.32)	<0.001	−0.75 (−1.15, −0.35)	<0.001	−0.86 (−1.23, −0.48)	<0.001	−0.61 (−0.93, −0.29)	<0.001
Model 3	−0.56 (−0.93, −0.18)	<0.001	−0.84 (−1.36, −0.32)	<0.001	−0.85 (−1.30, −0.40)	<0.001	−0.61 (−1.00, −0.23)	<0.001
**Female (*****n*** = **852)**								
Unadjusted	−0.79 (−1.23, −0.36)	<0.001	−0.79 (−1.42, −0.15)	0.02	−1.37 (−1.95, −0.79)	<0.001	−0.78 (−1.28, −0.28)	0.002
Model 1	−0.59 (−1.04, −0.14)	0.010	−0.53 (−1.17, 0.12)	0.2	−1.20 (−1.78, −0.62)	<0.001	−0.66 (−1.16, −0.16)	0.01
Model 2	−0.59 (−1.04, −0.14)	0.01	−0.55 (−1.19, 0.10)	0.1	−1.20 (−1.78, −0.62)	<0.001	−0.66 (−1.16, −0.16)	0.01
Model 3	−0.39 (−0.94, 0.17)	0.2	−0.49 (−1.40, 0.43)	0.3	−0.85 (−1.57, −0.14)	0.02	−0.44 (−1.09, 0.21)	0.2

Adjusted covariates: Model 1 = smoking + education + age.

Model 2 = Model 1 + body mass index.

Model 3 = Model 2 + (creatinine, uric acid, blood sugar, low-density lipoprotein, total cholesterol, C-reactive protein, Glycated hemoglobin).

Across all study subjects, those with possible sarcopenia were 1.40 (95% CI: 1.05, 1.87) times more likely to have cognitive scores decline than subjects without sarcopenia (Table 4). Subjects with possible sarcopenia were 1.41 (95% CI: 1.06, 1.87) times more likely to have a cognitive score below 11.5 than subjects without sarcopenia, and the difference was statistically significant. Further subgroup analysis by gender provided a similar result in males. Men with possible sarcopenia were 1.46 (95% CI: 1.02, 2.10) times more likely to have a cognitive score between 11.5 and 14 than men without sarcopenia. Individuals with possible sarcopenia were 1.51 (95% CI: 1.05, 2.16) times more likely to have a cognitive score lower than 11.5 points than those without sarcopenia, and the difference was statistically significant (*p* = 0.001). In females, the risk of low cognitive scores in those with possible sarcopenia was 1.69 times (95% CI: 1.17, 2.43) (*p* = 0.005) greater than that in those without sarcopenia. But after adjusting for blood-related variables in females (Model 3), no significant associations were observed.

**Table 4:** Association between Sarcopenia and cognitive function.

**Variables**	**Cognitive function scores**						***P-trend***
	**Highest tertile**		**Middle tertile**		**Lowest tertile**		
	***N***	**OR (95% CI)**	***N***	**OR (95% CI)**	***N***	**OR (95% CI)**	
**Overall**							
Unadjusted	785	1	759	1.65 (1.31, 2.09)	847	1.90 (1.52, 2.39)	<0.001
Model 1		1		1.52 (1.19, 1.93)		1.60 (1.27, 2.03)	<0.001
Model 2		1		1.51 (1.19, 1.93)		1.59 (1.26, 2.01)	<0.001
Model 3		1		1.40 (1.05, 1.87)		1.41 (1.06, 1.87)	<0.001
**Male**							
Unadjusted	487	1	511	1.80 (1.34, 2.42)	541	2.05 (1.54, 2.74)	<0.001
Model 1		1		1.59 (1.17, 2.16)		1.67 (1.24, 2.26)	<0.001
Model 2		1		1.59 (1.17, 2.16)		1.66 (1.23, 2.24)	<0.001
Model 3		1		1.46 (1.02, 2.10)		1.51 (1.05, 2.16)	<0.001
**Female**							
Unadjusted	298	1	248	1.44 (0.97, 2.12)	306	1.69 (1.17, 2.43)	0.02
Model 1		1		1.38 (0.93, 2.06)		1.46 (1.00, 2.13)	0.002
Model 2		1		1.37 (0.92, 2.04)		1.45 (0.99, 2.11)	0.003
Model 3		1		1.25 (0.79, 1.98)		1.30 (0.80, 2.10)	0.1

Adjusted covariates: Model 1= smoking + education + age.

Model 2 = Model 1 + body mass index.

Model 3 = Model 2 + (creatinine, uric acid, blood sugar, low-density lipoprotein, total cholesterol, C-reactive protein, Glycated hemoglobin).

## Discussion

We found that the presence or absence of sarcopenia in an older population may have a differential impact on cognitive performance. According to this cross-sectional study, among the older population in China, those with possible sarcopenia are at high likelihood of having low cognitive function than those without sarcopenia. The present study showed that the prevalence of possible sarcopenia in female subjects was higher than that in male subjects (27.46 vs. 27.16%); this difference may be due to the larger sample size of males than females in this study. The prevalence of sarcopenia was recently found to be 21.7 and 33.3% in females and males, respectively, but the population in that study was aged 80–99 years (27). According to the present study, older Chinese adults with possible sarcopenia are at greater risk of low cognitive function than those without sarcopenia, and sarcopenia and cognitive function are closely related. This finding is consistent with those of other studies (8, 28). However, recent findings regarding the relationship between sarcopenia and cognition are controversial. A study including 3,025 women over the age of 75 found no association between sarcopenia and cognitive impairment, regardless of adjustment for any underlying factors (29). In addition, a US study showed that sarcopenia was not associated with cognitive function in adults aged 60–69 but was associated with cognitive function in those over the age of 70 (30). The above discrepancies may be due to different diagnostic criteria for a cognitive decline and sarcopenia. In particular, in regard to assessment of cognitive function, there are many options, such as the Mini-Mental State Examination (MMSE) for the detection of MCI, the Montreal Cognitive Assessment (MoCA) and the Wechsler Adult Intelligence Scale (WAIS). This paper used cognitive scores, rather than grading scales, to understand the relationship between sarcopenia and cognitive function.

This article also examined the relationship between various components of sarcopenia and cognitive scores, finding that low muscle mass, low muscle strength, and slow pace (poor physical fitness) were all negatively correlated with cognitive scores. These results are consistent with those of other studies. For example, studies have shown that in older Chinese men, lower muscle mass was associated with higher depression scores (31). Several possible mechanisms may explain the association between cognitive impairment and sarcopenia. First, cognitive impairment often results in reduced physical activity (e.g., greater bed rest or a more sedentary lifestyle) and inadequate dietary intake, which may contribute to excessive muscle loss in older adults (32). Second, a mechanism shared by sarcopenia and cognitive impairment is inflammation. Age-related chronic low-grade inflammation characterized by elevated interleukin-6 (33) and tumor necrosis factor-α levels is also an important cause of sarcopenia and the development of cognitive impairment (34). Third, excessive oxidative stress associated with chronic diseases may lead to skeletal muscle atrophy (35) as well as muscle loss (36). Excessive oxidative stress also plays a crucial role in neuronal degeneration and cognitive impairment (37). Moreover, sarcopenia is often associated with loss of physical independence, frequent falls, and poor quality of life and results in decreased activity, which may result in reduced blood circulation to the brain, thus impairing cognitive decline (38).

In addition, the present study, which adjusted for sex, found that possible sarcopenia was significantly associated with low cognitive function in men and that participants with possible sarcopenia were at greater risk of having low cognitive function than those without sarcopenia. However, sarcopenia was not associated with low cognitive function in female in fully adjusted model. One possible reason for this difference may be the small sample size in females. In addition, the Partial Androgen Deficiency in Older Men (PADAM) study showed that older men experience a partial, gradual and variable decline in testosterone, manifested by depression, lack of motivation and energy, and lower mental vitality, with age (39). Sex hormone levels decrease with age and play an important role in the pathogenesis of age-related sarcopenia (40, 41). Moreover, the relationship between sarcopenia and depressive symptoms may be complicated by decreased levels of sex hormones (42). This also suggests that attention should be paid to effect of sex difference on low cognitive function of sarcopenia.

There are some limitations in this study. First, a significant amount of data in CHARLS is missing or incomplete, which could lead to biases. We also did a comparison of basic information between the excluded and included samples, there were still differences in their gender and age. However, we also found the average age of the included participants was 67.04 ± 5.57 years, which was similar to a previous study (68.13 ± 6.46 years) using the 2015 CHARLS data (21). Second, the items in the cognitive questionnaire only assessed cognitive status and could not diagnose the presence of cognitive impairment. Additionally, the cross-sectional design limited the ability to establish causality in the relationship between sarcopenia and cognitive function. Further longitudinal studies are needed to explore the causal relationship between sarcopenia and cognition. The choice of 2.5-m for the pace evaluation method was another limitation of our article. The original data used a 2.5-m walk to test gait speed rather than 6-m walk recommended by the AWGS 2019. Another study concluded that “distance walked during the gait speed test did not influence the recorded gait speed” (43). Thus, 2.5-m walk may be suitable for the walking-pace assessment of older Chinese adults. On the other hand, the present study has many strengths. First, this study analyzed CHARLS data. CHARLS data is nationally representative, and thus our findings reflect the relationship between sarcopenia and cognitive status older Chinese adults. Second, this study analyzed people with possible sarcopenia and provides recommendations for those who do not meet the criteria for sarcopenia diagnosis. Third, as the study used a cross-sectional analysis, there were naturally occurring contemporaneous controls in the sample that were comparable.

The present study found that sarcopenia and low cognitive function were correlated in older individuals and that sarcopenia is a risk factor for low cognitive function in older individuals. Thus, sarcopenia prevention and treatment can be used as a therapeutic measure to prevent or delay low cognitive function in older individuals, to control and manage sarcopenia or reduce the risk of sarcopenia in high-risk groups, and to enable early detection and treatment. Sarcopenia prevention and treatment can reduce low cognitive function in older individuals; prevent cognitive impairment; reduce the burden on individuals, families and society; improve the living standards of older individuals; and provide new methods and ideas for improving the health of older individuals.

## Data availability statement

The datasets presented in this study can be found in online repositories. The names of the repository/repositories and accession number(s) can be found in the article/supplementary material.

## Ethics statement

The studies involving human participants were reviewed and approved by Ethics approval for the CHARLS study was obtained from the Institutional Review Board (IRB) at Peking University. The IRB approval number for the main household survey was IRB00001052-11015 and for the biomarker collection was IRB00001052-11014. The patients/participants provided their written informed consent to participate in this study.

## Author contributions

HD designed the study and wrote the paper. MY performed the statistical analyses. HX revised the manuscript. XL collected and interpreted data. YC contributed to the interpretation of study results. ZL contributed to acquisition of funding, study design, and provided administrative support. All authors have read and agreed to the published version of the manuscript.
